# 

**Published:** 1899-10

**Authors:** 


### CONTENTS.

### COMMUNICATIONS.

The Relative Toxity of Cocain and Eucain .......... 445
Professional Ethics .......... 451
Syphilitic Infection .......... 454
Diseases of the Antrum of Highmore; a Study of One Hundred and Fifty Cases .......... 459

### PROCEEDINGS.

National Dental Association, Niagara Falls, August 1–4, 1899 .......... 463
The Specialization of Our Preliminary Education .......... 466
Dental History .......... 469
Dental Articulation and Occlusion .......... 470

### SELECTIONS.

Changes in the Salivary Glands in Diabetes .......... 472
The Value of Concentrated Foods .......... 473
High Altitudes and the Blood Count .......... 475
Training the Hand .......... 476
Schemes for Hastening the Millennium .......... 477
Contagion and Infection .......... 477
The Dental Board of Victoria .......... 478
A Movement has been Started .......... 478
As Others See Us .......... 479
By the Will of the late Dr. Chas. J. Stille .......... 479
A Keen Diagnosis .......... 480
Temperance Restaurants .......... 480
Plaster of Paris .......... 480
Eczema of the Lips Caused by Mouth Washes and Tooth Powders .......... 481
Local Anesthesia .......... 481
Earache .......... 481
Barbaric Dentistry .......... 482
The Cold Morning Bath .......... 482
Examining Michigan Recruits .......... 482
The Board of Health of the State of Rhode Island .......... 483
When Russia Takes any Matter in Hand .......... 483
The Invention of Spectacles .......... 483
Bacteriologic Enzymes as the Cause of Acquired Immunity .......... 484
Physiology of Grafting .......... 484
Death by Chloroform .......... 484
To Keep the Hands White and Soft .......... 485
An Enforced Anti-Spitting Law .......... 485
The Standard Preliminary to a Medical Education .......... 486
A Source of Riches .......... 486
Iodoformogene .......... 486
Growth of Human Hair .......... 486
Pulp Capping .......... 487
The Anthrax Bacillus Does Not Form Toxins .......... 487
Remarkable how Much Salt Water a Colon will Absorb .......... 487
A Supreme Court Decision .......... 487

### NOTICES.

Union Meeting Seventh and Eighth District Dental Societies, State of New York .......... 488
Examinations .......... 489

### EDITORIAL.

Special Interest to the Dental Profession .......... 490
Pennsylvania State Dental Society .......... 492
Ohio State Dental Society—Annual Meeting .......... 492

